# Factors associated with patient and health system delays in diagnosis and treatment of tuberculosis in Montenegro, 2015–2016

**DOI:** 10.1371/journal.pone.0193997

**Published:** 2018-03-09

**Authors:** Olivera Bojovic, Milic Medenica, Danko Zivkovic, Bozidarka Rakocevic, Goran Trajkovic, Darija Kisic-Tepavcevic, Anita Grgurevic

**Affiliations:** 1 Department for Tuberculosis, Hospital for Lung Disease and Tuberculosis Brezovik, Niksic, Montenegro; 2 Faculty of Medicine at the University of Montenegro, Podgorica, Montenegro; 3 Department of Pulmonary Internal Medicine at Clinical Hospital Center, Podgorica, Montenegro; 4 Center for Disease Control and Prevention, Institute of Public Health, Podgorica, Montenegro; 5 Institute of Medical Statistics and Informatics, Faculty of Medicine, University of Belgrade, Belgrade, Serbia; 6 Institute of Epidemiology, Faculty of Medicine, University of Belgrade, Belgrade, Serbia; Indian Institute of Technology Delhi, INDIA

## Abstract

**Background:**

Fundamental measures of control of tuberculosis are early detection and timely treatment of the affected. The aim of this study was to identify factors associated with patient-related and health system-related delays among patients with tuberculosis in the Republic of Montenegro.

**Methods:**

A cross-sectional study included 130 tuberculosis patients older than 15 years of age. The inclusion criteria were diagnosis of tuberculosis based on clinical, pathohistological and microbiological findings. Patient delay referred to the number of days between the onset of symptoms and the first consultation with general practitioner (GP). Health system delay represented the number of days between the first consultation with GP and the initiation of tuberculosis treatment.We classified delays longer than median delay length as 'prolonged delays'. Delays greater than 75^th^ percentile of the maximum length of delay were classified as 'extreme delays'.

**Results:**

Distribution of patient and health system delay in the overall delay was apprioximately equal (49% vs. 51%). Being married (OR = 2.54, p = 0.026) and having more negative attitudes towards tuberculosis (OR = 4.00, p = 0.045) were associated with extreme patient delay. Greater knowledge on tuberculosis was associated with lower likelihood of prolonged (OR = 0.24, p = 0.031) and extreme (OR = 0.30, p = 0.012) patient delay. Persons with negative sputum smear were more likely to experience prolonged (OR = 7.01, p<0.001) and extreme (OR = 4.40, p = 0.032) health system delay. Persons older than 47 years of age were more likely to experience prolonged health system delay (OR = 2.61, p = 0.042). Specialist consultation delay was associated with prolonged (OR = 1.08, p = 0.001) and extreme (OR = 1.05, p<0.001) health system delay.

**Conclusion:**

Contribution to overall delay is equally distributed between the patients and the health care system. Improvement of knowledge in the general population and continuing medical education of the health care workers on tuberculosis could lead to reduction in patient and health system delays in treatment of tuberculosis.

## Introduction

Despite considerable improvements in controlover the past two decades, tuberculosis remains a significant public health problem as well as the leading cause of death from infectious diseases worldwide[1,2].The World Health Organization (WHO) estimatedthat a total of 10.4 million people developed and 1.6 million people diedfrom tuberculosis in 2016[2].Fundamental measures of control of tuberculosis are early detection and timely treatment of the affected. Contact with undetected, infectious patientsfacilitates further transmission of tuberculosis in the general population. The duration of infectiousness of a tuberculosis patient is essential for increase inexposure risk of the general population [3].

Therefore, delays in detection and initiation of tuberculosis treatment can be barriers to effective disease control. Delays are common in both developed and developing countries as well as in countires with low or high tuberculosis incidence, either due to delay in seeking health care (patient delay) or delay inreaching thediagnosis (health system delays) [4–7]. Regardless of the type, consequences of delays remain similar: poorerprognosis and spread of infection in the population. Because of this, it is important to assess the length and significance of both types of delay and to analyze the reasons as to why delays occur in order to tailor appropriate and efficient interventions.

There has been no consensus at the national or international level on the acceptable length of delay from the onset of symptoms to treatment initiation. It is indispensablefor acertain country toidentify the determinants of delays, given specific social and cultural backgrounds as well as specific national programs, to improve the quality and efficiency of the national tuberculosis control program [8–10].

Numerous studies have examined the matter of diagnostic and treatment delays, mainly in countries with high or low tuberculosis incidence [4–7, 9, 11]. Nevertheless, studieson this issue in countries with medium incidence, such as Montenegro,are rather limited [12]. The Republic of Montenegro, located in Southeast Europe, has a population of 620,029 inhabitants and reported tuberculosis incidence rate of 14/100,000 in 2016. This incidence exhibits a slow,butsteady decline over the past 15 years [13]. The National Tuberculosis Control Program has been integrated into the country's health care system and ensures diagnosticprocedures and treatment of tuberculosis for all citizens free of charge. Persons with complaints first seek their chosen physician (general practitioner (GP))at the primary health care institutions. In settings where tuberculosis is not as common, lack of awareness and suspicion of tuberculosis among both patients and physicians at the primary health carelevel have been recognized as the weak link in the Program [14].

Data on patient and health system delays as well as factors associated with delays in the Republic of Montenegro are lacking. In line with Pillar No. 3 (instensified research and innovation) of the Global strategy for prevention, care and control of tuberculosis after 2015 [15], this study could contibute to further strengthening of the health care system, placing particular emphasis on the primary health care delivery.

The aim of this study was to identify factors associated with patient-related and health system-related delays among patients with tuberculosis in Montenegro.

## Materials and methods

### Study design and participants

This research was performed using a cross-sectional study design. The study included all patients treated for tuberculosis in the Specialized Hospital for Lung Diseases in Brezovik, Niksic, from January 1, 2015 to Jun 30, 2016. This Hospital is the reference institution for tuberculosis treatment in the Republic of Montenegro. The inclusion criteria were as follows: diagnosis of tuberculosis based on clinical, pathohistological and microbiological findings, age above 15 years and signed informed consent for participation in the study. Persons who had poor general health status and were unable to answer questions as well as persons who had severe psychiatric disorders were excluded from the study.

### Instruments

Data were collected by using a questionnaire based upon similar studies in other populations [3,16]. The questionnaire consisted of 53 questions divided in four segments. First segment comprised items on participants' demographic characteristics (age, gender, place of residence, marital status), socio-economic data (education level, employment status, monthly income), living conditions, number of household members and their age and information on previous contacts with tuberculosis patients. Second segment referred to knowledge on tuberculosis relative to etiology, transmission, symptoms, most commonly affected organ, treatment and prevention. The level of knowledge was calculated based on median number of correct answers (score below median = low level of knowledge; score above median = high level of knowledge). Each correct answer was awarded one point. The sum of each point per item represented the knowledge score. The score ranged from 0 to 20.Third segment comprised items on attitudes of the participants towards tuberculosis. These items examined attitudes towards population groups at risk and medical conditions as risk factors for development of tuberculosis, feelings of shame, concealment of diagnosis, actions of family and friends, social repercussion for the family and voluntary work at the community with a purpose to share personal experiences. The attitude level was calculated based on median (score below median = more positive attitudes; score above median = more negative attitudes). Each answer was awarded one point. The sum of each point per item represented the attitude score. The score ranged from 0 to11. Fourth segment in the questionniare was related to items assessing patient delays (time from onset of symptoms until the initiation of treatment) and health system delays at various levels of health care delivery (GPs, pneumophtysiology service) as well as the overall delay. Other data comprised bacteriological confirmation of tuberculosis, staging of disease at the time of diagnosis, physical findings and number of the affected resulting from contact with the patient and chemoprevention.

Patients were interviewed at the end of the initial treatment phase, before hospital discharge to eliminate potential data sharing between patients. After each wrong answer in the knowledge segment, the interviewer provided explanations and educational material. Patients were also given an end-of-hospitalization questionnaire with similar questions on knowledge of tuberculosis (not included in the present study). Data on diagnostic procedures and initiation of treatment were validated in patients' medical records (microbiological and pathohistological reports and tuberculosis medical records).

The Ethical Committee of the Faculty of Medicine, University of Belgrade, reviewed and approved the study.All participants provided signed informed consent before enrolment. Signed informed consents for the two participants under the age of 18 wereobtained by their parents.

### Definitions

Patient delay referred to the number of days between the onset of symptoms and the first consultation with GP. In case of diverse complaints, we accounted the longest lasting symptom.

Health system delay represented the number of days between the first consultation with GP and the initiation of tuberculosis treatment. The actual date of diagnosis seldom differed from treatment initiation date. In those medical records where the date of diagnosis was omitted, we considered the date of treatment initiation.

Specialist consultation delay was defined as the period between the first consultation with GP and the first consultation with the pulmonary specialist.

Because the standardized definitions are lacking, we classified delays longer than median delay length as 'prolonged delays'. Also, delays greater than 75^th^ percentile of the maximum length of delay were classified as 'extreme delays'.

### Statistical analyses

Data were analyzed using descriptive statistics and logistic regression models. Univariate logistic regression analysis was performed to determine the association between each explanatory variable and prolonged or extreme patient/health system delay in the initiation of tuberculosis treatment. All variables univariately associated with the outcome at the level of significance P < 0.1 were entered into the final model of multiple logistic regression. Computed measures of independent contribution to patient/health system delay were the adjusted odds ratios (OR). Variables from the multiple logistic regression analysis at the significance level of P<0.05 were considered as independent factors associated with prolonged and extreme delays.

## Results

The study included 130 tuberculosis patients. Tuberculosis was confirmed using direct microscopy in 76 cases (58.5%), culture in 97 cases (74.6%) and pathohistological specimens in 15 cases (11.5%). In 18 cases (13.9%) the disease was diagnosed using radiography and signs and symptoms suggestive of tuberculosis, while other lung diseases were ruled out in additional diagnostic procedures. In the study sample, there were 83 males (64%) and 47 females (36%). Age of the participants ranged from 16 to 85 years with median of 47 years. Basic demographic, socioeconomic and clinical characteristics of study participants are presented in Table 1.

**Table 1:** Characteristics of TB patients.

Variables	Number (%)
**Gender**	130 (100)
Male	83 (63.8)
Female	47 (36.2)
**Age at diagnosis (years)**	
≤ 47	65 (50)
> 47	65 (50)
**Place of residence**	
Rural	30 (23.1)
Urban	100 (76.9)
**Housing conditions**	
Own house/flat	106 (81.5)
Tenant	21 (16.2)
Refugee	3 (2.3)
**Highest level of educational attainment**	
No formal education	9 (6.9)
Elementary school	21 (16.2)
Secondary school	79 (60.8)
Higher education	21 (16.2)
**Employment status**	
Employed	47 (36.2)
Unemployed	83 (63.8)
**Marital status**	
Married	68 (52.3)
Other (single,divorced, widower)	62 (47.7)
**Contact with TB patient**	
Yes	43 (33.1)
No	87 (66.9)
**Point of Contact**	
Household	25 (19.2)
In the family	12 (9.2)
At work/college/school	2 (1.5)
Other	4 (3.1)
**Personal monthly income^a^**	
Average and above average	30 (23.1)
Below average	100 (76.9)
**TB knowledge score**	
Low degree ≤ 17	67 (51.5)
High degree >17	63 (48.5)
**Attitudes score**	
Low degree ≤ 5	55 (42.3)
High degree >5	75(57.7)
**Frequency of TB symptoms**	
Cough	89 (68.5)
Night sweats	55(42.3)
Loss of appetite	64 (49.2)
Weight loss	68 (52.3)
Hemoptysis	10 (7.7)
Fever	61 (46.9)
Chest pain	41 (31.5)
Other non tuberculosis symptoms	34 (26.2)
**Number of TB symptoms**	
≤ 1	32(24.6)
> 1	98(75.4)
**Comorbidity**	
Yes	41 (31.5)
No	89 (68.5)
**Previous hospitalisation**	
Yes	39 (30.0)
No	91 (70.0)
**Tuberculosis diagnosis reached**	
Bacteriologically	97 (75.0)
Histologically	15 (11.0)
Clinically	18 (14.0)
**Sputum smear**	
Smear-positive	76 (58.5)
Smear-negative/culture positive	21 (16.2)
Smear negative /culture negative	33 (25.3)
**Cavity on chest radiography**	
Yes	77 (59.2)
No	53 (40.8)

^a^According to the Bureau of Statistics- Montenegro.

Median length of patient delay was 30 days (length range 0–1095 days). The 75^th^ percentile of the total lengthof patient delay was 85 days. Median length of health system delay was 27 days (length range 1–736 days), while 75^th^ percentile of the total length of health system delay was 77 days. Median length of overall delay, was 84 days (overall delay range 4–1159 days), and 75^th^ percentileof the total length of overall delay was 160 days.

### Patient delay

To elucidate factors associated with patient delay, we analyzed participants' characteristics shown in Table 1. Univariate logisitic regression models did not show associationsof demographic, socioeconomic or clinical characteristics with prolonged delay. However, participants' knowledge level and experiencing prior weight loss were univariately associated with prolonged delay at the probability level of p < 0.1. These two variables entered the multiple regression model. This model showed association between prior weight loss and long patient delay (OR = 3.01, p = 0.046), while persons who had higher knowledge level on tuberculosis were less likely to experienceprolonged delay (OR = 0.24, p = 0.031) (Table 2). Additionally, according to multiple logistic regression model, being married (OR = 2.54, p = 0.049) and unfavorable attitude towards tuberculosis (OR = 4.00, p = 0.045) were associated with extreme delay. Also, persons who were more knowledgeable of tuberculosis were less likely to experience extreme delays (OR = 0.30, p = 0.012).

**Table 2:** Factors associated with prolonged and extreme patient delay: Results of logistic regression analysis.

Variable	Prolonged delay				Extreme delay			
	Univariate logistic regression		Multiple logistic regression^c^		Univariate logistic regression		Multiple logistic regression^c^	
	OR (95% CI)	p-value	OR (95% CI)	p-value	OR (95% CI)	p-value	OR (95% CI)	p-value
TB knowledge score ^b^: Low degree <17 *vs.High degree ≥17	0.30 (0.09–1.02)	0.054	0.24 (0.07–0.88)	0.031	0.35(0.15–0.82)	0.016	0.30(0.17–0.77)	0.012
TB symptoms: Weight loss	2.45(0.88–6.78)	0.084	3.01(1.02–8.90)	0.046				
Attitudes score ^b^: Low degree ≤ 5 vs. High degree >5 *					4.29(1.21–15.19)	0.024	4.00(1.03–15.50)	0.045
Comorbidity: Yes vs. No*					2.05 (0.89–4.68)	0.090	2.19 (0.89–5.40)	0.089
Marital status: Married vs. Other *					2.07(0.90–4.75)	0.086	2.54(1.01–6.41)	0.049

OR—Odds ratio; 95% CI—confidence interval.

^b,^Median cut-off.

^c^All variables associated with delays at a level of p < 0.1 were included in the multiple logistic regression analysis.

*Reference category.

### Health system delay

Median length of specialist consultation delay was 7 days, while 75^th^ percentile of the total length of specialist consultation delay was 28 days. Standardized guidelines for diagnosing tuberculosis include lung radiography and sputum smear and culture. Rapid lung radiography is anticipated for persons suspective of having tuberculosis, thus median period of waiting for chest X-ray was 1 day, while 75^th^ percentile of the delay length for this procedure was 12 days. Median delay for dispatch of sputum specimen was 15 days (75^th^ percentile of delay length was 41 days). Given that tuberculosis therapy is initiated usually on the same day when the disease is diagnosed (median delay 0 days, 75^th^ percentile of delay was 2 days), the date of treatment initiation was considered as the end-point of this analysis.

Univariate logistic regression models showed that the following characteristics were associated with prolonged and extreme health system delay: persons above 47 years of age, negative sputum smear and culture, longer delays for undergoing a chest X-ray after initial GP consultation and longer specialist consultation delay. Being older than 47 years was associated with both prolonged (p = 0.024) and extreme health systemdelay (p = 0.045). With each day of delayed chest radiography the risk of prolonged and extreme health system delay increased by 5% and 4%, respectively. Similarly, each day of specialist consultation delay was associated with a 7% (p<0.001) increase in risk for prolonged health system delay and with a 5% (p = 0.001) increaseinrisk for extreme health system delay. Negative sputum smear was associated with prolonged (p<0.001) and extreme health system delay (p = 0.001) (Table 3).

**Table 3:** Factors associated with prolonged and extreme health system delay: Results of logistic regression analysis.

Variable	Prolonged delay				Extreme delay			
	Univariate logistic regression		Multiple logistic regression^c^		Univariate logistic regression		Multiple logistic regression^c^	
	OR (95% CI)	p-value	OR (95% CI)	p-value	OR (95% CI)	p-value	OR (95% CI)	p-value
Age range(years)^b^: > 47 vs. ≤ 47*	2.25 (1.11–4.54)	0.024	2.61 (1.03–6.56)	0.042	2.34 (1.02–5.38)	0.045		
Sputum smear: Smear negative vs. positive*	6.83 (3.10–15.05)	<0.001	7.01 (2.71–18.08)	<0.001	4.54 (1.92–10.70)	0.001	3.40 (1.11–10.38))	0.032
Specialist consultation delay (days)	1.07 (1.03–1.11)	<0.001	1.08 (1.03–1.13)	0.001	1.05 (1.03–1.07)	<0.001	1.05 (1.03–1.07)	<0.001
Chest X-ray delay (days)	1.05(1.02–1.09)	0.002			1.04(1.02–1.06)	<0.001		
Bakteriological diagnosis: Smear—and Culture- vs.Smear ± or Culture+*	5.52 (2.18–13.97)	<0.001			4.77 (2.00–11.35)	<0.001		
Comorbidity: Yes vs. No*					2.92 (1.28–6.69)	0.011		
Number of TB Symptoms: >1 vs. 1*					0.24 (0.10–0.57)	0.001		

OR-odds ratio; 95% CI—confidence interval.

^b^ Median cut-off.

^c^All variables associated with delays at a level of p < 0.1 were included in the multiple logistic regression analysis.

*Reference category.

According to multiple regression model, negative sputum smear was independently associated with prolonged (OR = 7.01, p<0.001) and extreme (OR = 3.40, p = 0.032) health system delay.Each day of specialist consultation delay increased the likelihood of prolonged health system delay by 8% (OR = 1.08, p = 0.001) and extreme health system delay by 5% (OR = 1.05, p<0.001). Being older than 47 years of age was associated with prolonged health system delay (OR = 2.61, p = 0.042) (Table 3).

## Discussion

The results of this study showed that the median length of patient and health system delay in Montenegro was 30 and 27 days, respectively. Additionally, extreme delay, based on the value of the 75^th^ percentile of maximum delay length, accounted for 85 days among patients and for 77 days in the health system. Therefore, overall delay accounts for 49% of patient delay and 51% of health system delay. In systematic review of delay with similar distribution of delays between patients and health system[9], patient delays ranged from 18 to 43 days, while health system delays ranged from 13 to 39 days. However, differences between our and previous studies may be attributed to inconsistent reporting of tuberculosis as well as to diverse level of socioeconomic development.

Causes of patient delay are suggested to be complex. Reported reasons might be different level of health literacy, ambiguous attitudes towards physicians, ability to recognize early symptoms of the diseaseor differences in access to health care [8].

In this study, we observed several factors associated with patient delay. In terms of demographic characteristics, marital status was associated with extreme patient delay. Previous study conducted in Ethiopia reported longer patient delays among persons who were married, however, more detailed explanation for this association was lacking[17]. In fact, potential reason for such a result could be cultural factors affecting health care access. This, on the other hand, is not applicable to Montenegro, as health care is equally accessible to all the residents. Given that economy of Montenegro is presently in transition, development of various health conditions is likely one out of many issues that people in such economies encounter. It is possible that challenges related to provision for families could be the reason for delayed decision to make an appointment with a physician.

Contrary to some studies [9], in our population, none of the socioeconomic factors were associated with prolonged or extreme patient delay. Diagnostic procedures and treatment of tuberculosis in Montenegro are free of charge, regardless of whether or not a person has health insurance. In this way, the national policy aims at reducing potential barriers to health care access, especially among underprivileged population groups. Similar policy has been enforced in the countries from this particular region, such as Croatia [16].

Tuberculosis has previously been a major health problem in our population. Therefore, it is not surprising that persons with higher knowledge level of tuberculosis were less likely to experience patient delay in our study. Being more knowledgeable, however, has not consistently been associated with lower likelihood of patient delay [18]. Organized effors in public health sector to raise awareness and knowledge on tuberculosis issue, particularly among vulnerable populations, appear to have been successful.

More negative attitudes towards tuberculosis, on the other hand, result in concealment of patients' health condition from their immediate surrounding, which in turn contributes to extreme patient delay. This could be explained by underlying stigmatization of tuberculosis, which has persisted as a cultural attribute in this region over the past century. Fear of diagnosis and isolation from immediate family has often been the cause of delayed physician visits. In some regions, fear has been underlined as important predictor of patient delay [3, 19]. Health education of the population and raising awareness of tuberculosis could help communities modify their beliefs and stigma surrounding this disease.

In accordance with previous reports [7, 9, 20], most common symptoms in our patients were cough, weight loss, loss of appetite and fever. Of all the reported symptoms, weight loss has been observed as a factor associated with patient delay. Potential reasons for this finding could be the circumstance that cough also occurs in later stages of the disease and could be, in fact, a result of delayed visit to GP. However, neglect of symptoms suggestive of certain non-communicable diseases with poor outcome, such as diabetes of cancer, could lead to disease progression and subsequent increase in mortality [12].

In our study, health delay refers, by and large, to delays in diagnostics, given that median time for delay in treatment was, in fact 0 days. We observed that health system delay and patient delay almost equally contribute to overall delay (51% vs. 49%, respectively). This is not satisfactory, given that other countries, such as Nepal or Croatia, report remarkably lower proportions of health system delay (26% and 30%, respectively). Broad network of 15 ambulatories specialized in lung diseases at the primary health care level cover the entire territory of Montenegro, which contrasts previuos reports of reduced access to health care [21, 22]. Nevertheless, in Montenegro it is customary that the first examination of persons aged above 15 years is performed by the chosen physican, and the examination must previously be appointed, at both, chosen physican and at lung disease ambulatory. It has been suggested [17, 23, 24]that repeated visits to the same, occasionally not suffiently experienced, physicianin the primary health care institutionsthat often lack adequate diagnostic capacity (either in public or privately owned facilities) contribute to health system delay. These cirucumstancescould partially explain our findings. In addition, longer specialist consultation delays were associated with prolonged and extreme health system delay. In our health system, it takes a median of 6 days, for a patient suspective of tuberculosis to transfer from GP to lung specialist, who further dispatches samples for microbiological analysis. This is not satisfactory compared with 3-day delays in the health system of Nepal [18]. In regions such as Montenegro, where tuberculosis is not as common, it is indispensible to have experienced and qualified staff, as well as to improve the level of alertness for tuberculosis among primary health care physicians.Reports of the Institute of Public Health of Montenegro suggested that almost one half (48%) of chosen physicians in the primary health care institutions did not see any patient who had tuberculosis, while a total of 17.5% examined one patient with tuberculosis over the past two years [25]. Because of this, it is mandatory to include continuing education of primary health care physicians on tuberculosis, in order to reduce health system delays.

Lung tuberculosis with positive sputum smear was reported in 58.5% of participants. Having negative sputum smears has been associated with prolonged and extreme health system delay, indicating weaknesses in the overall detection of tuberculosis. One reason for such an association could be the circumstance that only one reference laboratory for microbiologic testing of tuberculosis exists in the entire country and requires transportation of specimens from other parties. Delays in dispatching sputum samples (median 15 days, 75^th^ percentile 41 days) and undertaking other additional and complex diagnostic procedures for tuberculosis patients with negative sputum smears, could also contribute to health system delays. Development of laboratory capacities and accessibility of microbiological testing for rapid diagnostics of tuberculosis in routine practice could remedy this problem in our health system [26, 27].

We observed that persons who were older than 47 years of age were more likely to experience health system delays. Our results are in line with astudy from Australia reporting that compared to younger people, persons older than 45 years were likely to be diagnosed with tuberculosis at later stages [28]. The same study suggests that, compared to younger population, older persons were less likely to seek for a physician because of respiratory complaints, cough, hemoptysis and classic constituional symptoms.One of the possible explanations could be that in the elderly, respiratory problems such as caugh are less pronounced, which makes it difficult to obtain samples and bacil isolation, and thus a diagnosis[7]. Also, age-associated comorbidities and problems in differential diagnostics could be attributed to delays in older population [29, 30]. The higher incidence of other diseases (pneumonia, asthma, bronchitis, lung cancer) in relation to tuberculosis in Montenegro[13], increases the possibility of diagnostic errors due to low suspicion of tuberculosis in older age groups.

### Study limitations

Certain limitations of this study need to be addessed. Firstly, limitations of cross-sectional study design may have potentially biased our results, primarily because of wide range of confounding factors. Although we have controlled for confounding in the multiple logistic regression models, we cannot exclude the residual confounding factor effect. Therefore,associations observed in our study should be interpreted carefully and, due to study design, we are limited wheninferring causality.Secondly, some patients were unable to recall exact dates of onset of tuberculosis symptoms. In an effort to remedy this issue, we did not recruit consecutive patients for our study and we carried out in-depth interviewes with participants to prevent missing answers. Moreover, to validate data received from the patients we cross-checked all tuberculosis-related information in the corresponding medical records. All participants were interviewed by experienced and qualified physicians who conducted thorough interviews to elucidate exact onset of symptoms in order to reduce potential information bias. Despite these limitations, our study also has some advantages. The study sample is representative of the population who had tuberculosis, because all the treated patients were registered at the one reference tuberculosis center in the country. As such, our results could be generalized to the entire population of Montenegro.

## Conclusion

Because of ongoing decreasing tendency of tuberculosis incidence rates and its numerous non-specific signs and symptoms, tuberculosis is not prioritized as differential diagnosis at the primary health care level. Therefore, diagnostic delays are obvious and expected. On the other hand, long and extreme delays are not satisfactory, because these elements facilitate the spread of infection and render elimination of tuberculosis difficult to achieve. Contribution to overall delay is equally distributed between the patients and the health system. For this reason, both of these aspects need to be improved to enable early detection and treatment of tuberculosis. Improvement of knowledge on tuberculosis in the general population with emphasis on prevention, early detection and treatment as well as raising awareness among health care workers through continuing medical education on tuberculosis could lead to reduction in patient and health system delays.

## Supporting information

S1 Dataset Data of TB patients. (XLSX)
